# Supplementary material for: Clinically applicable histopathological diagnosis system for gastric cancer detection using deep learning
Source: Nat Commun. 2020 Aug 27;11:4294. doi: 10.1038/s41467-020-18147-8 (PMC7453200; doi:10.1038/s41467-020-18147-8)
Supplement: Supplementary file 2 — Supplementary Information [file 41467_2020_18147_MOESM2_ESM.pdf]

Clinically applicable histopathological diagnosis system for gastric cancer detection using deep learning

Song et al.

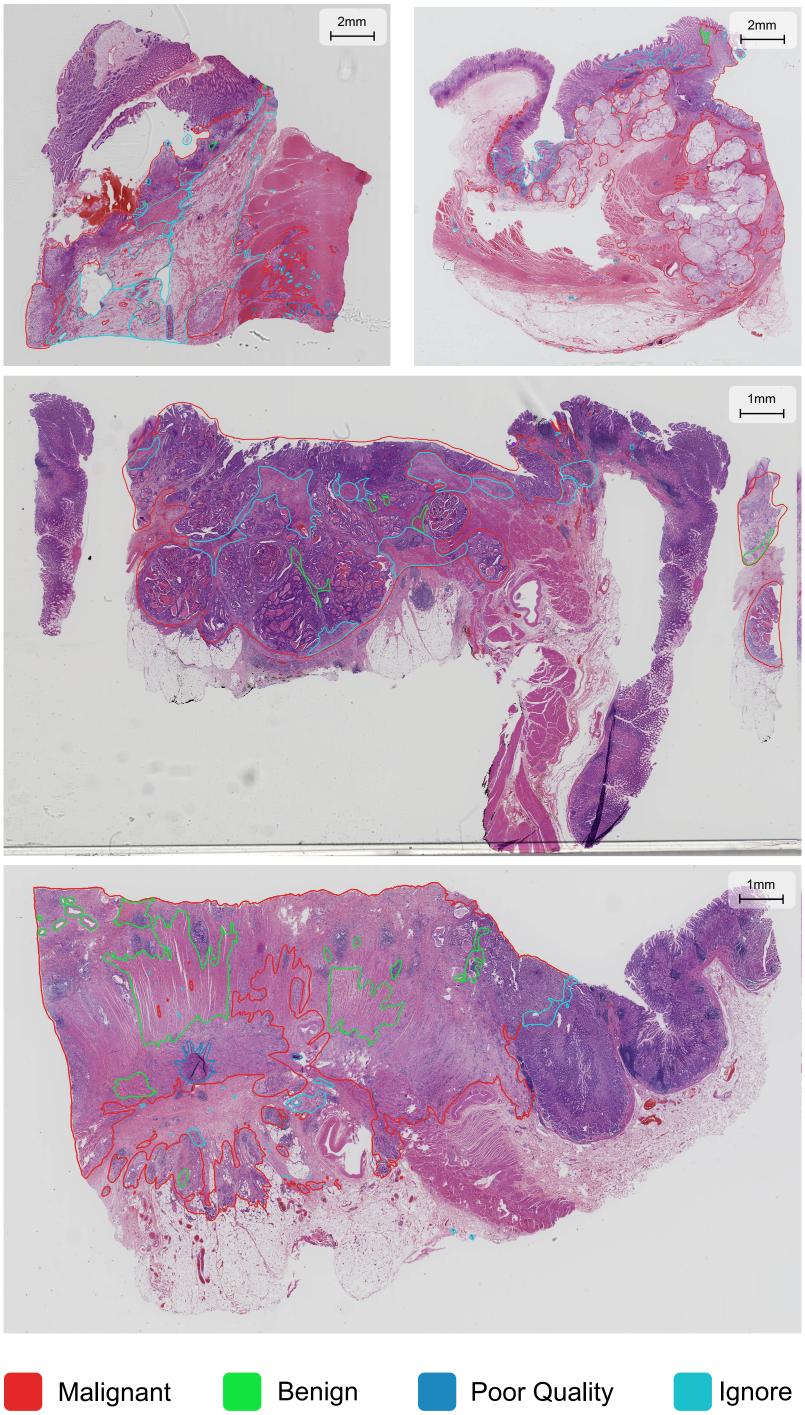

Figure 1: **Four examples of labelled WSIs.** The WSIs underwent the three-step annotation procedure. We obtained similar annotation results repeating the procedure three times.

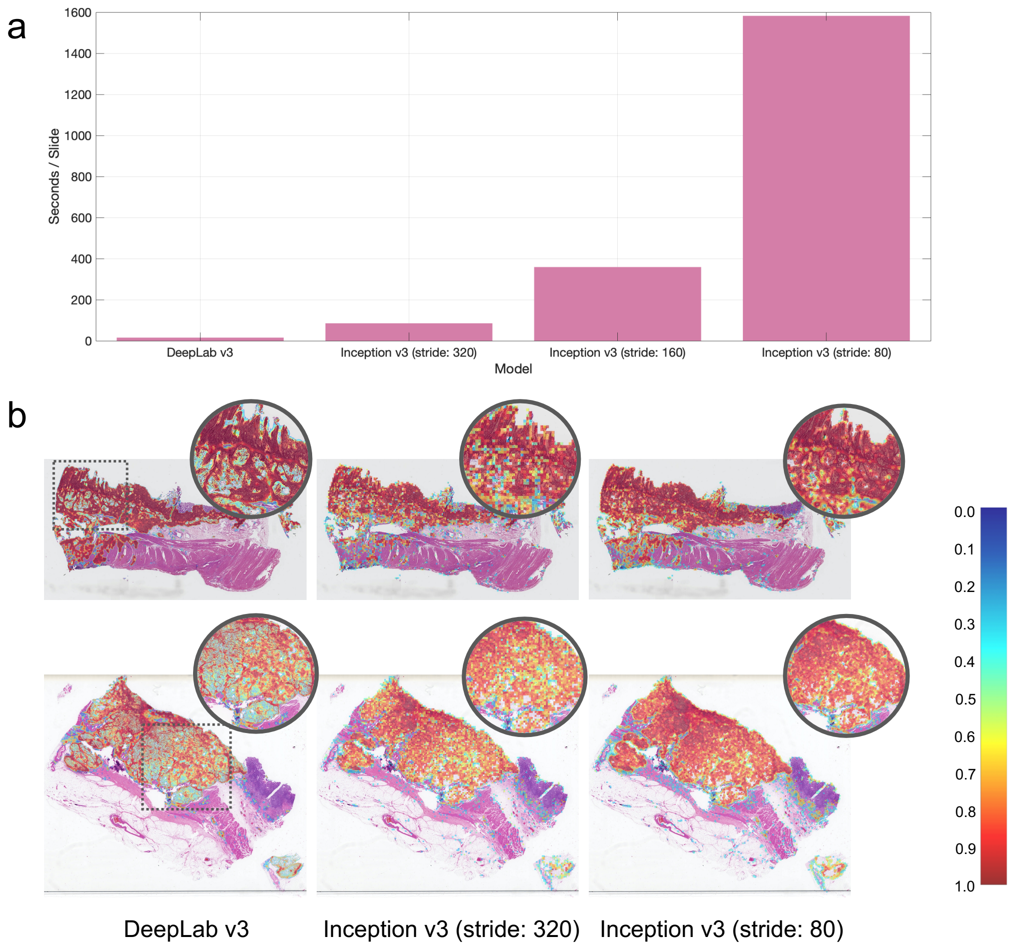

Figure 2: **Comparison between segmentation (DeepLab v3) and classification (Inception v3) models. a**, The average prediction time for 10 slides with size around 500 MB (with 4 GPUs). For the segmentation model, in the inference stage, we used tiles of $2,000 \times 2,000$ pixels and a 10 percent overlap ratio. For the classification model, we used tiles of size 320×320 pixels with different strides. **b**, Several predicted heatmaps from the models, we could see that the segmentation model reveals more interpretable predictions.

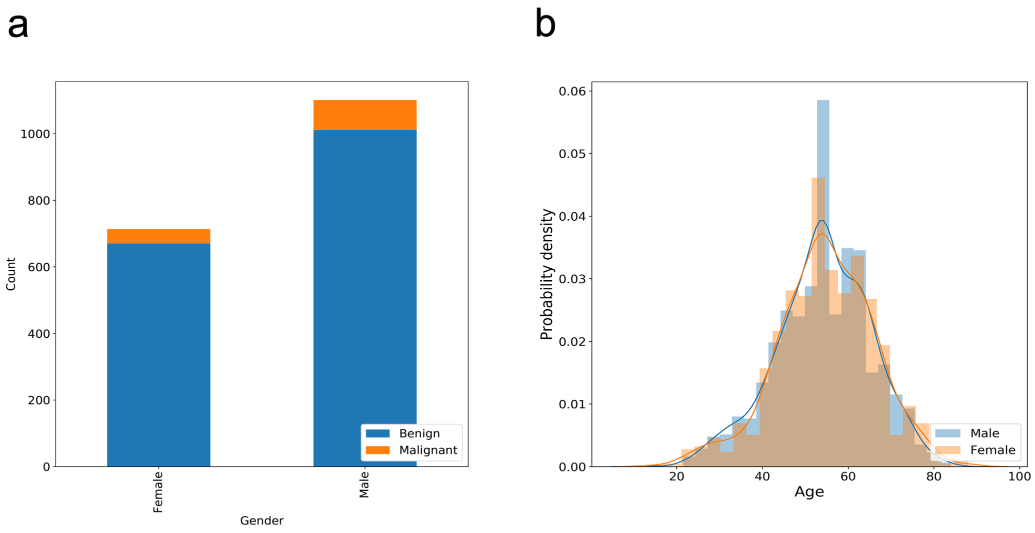

Figure 3: **Patient-level data distribution of the daily gastric dataset.** **a**, Patient gender distribution. **b**, Patient age distribution.

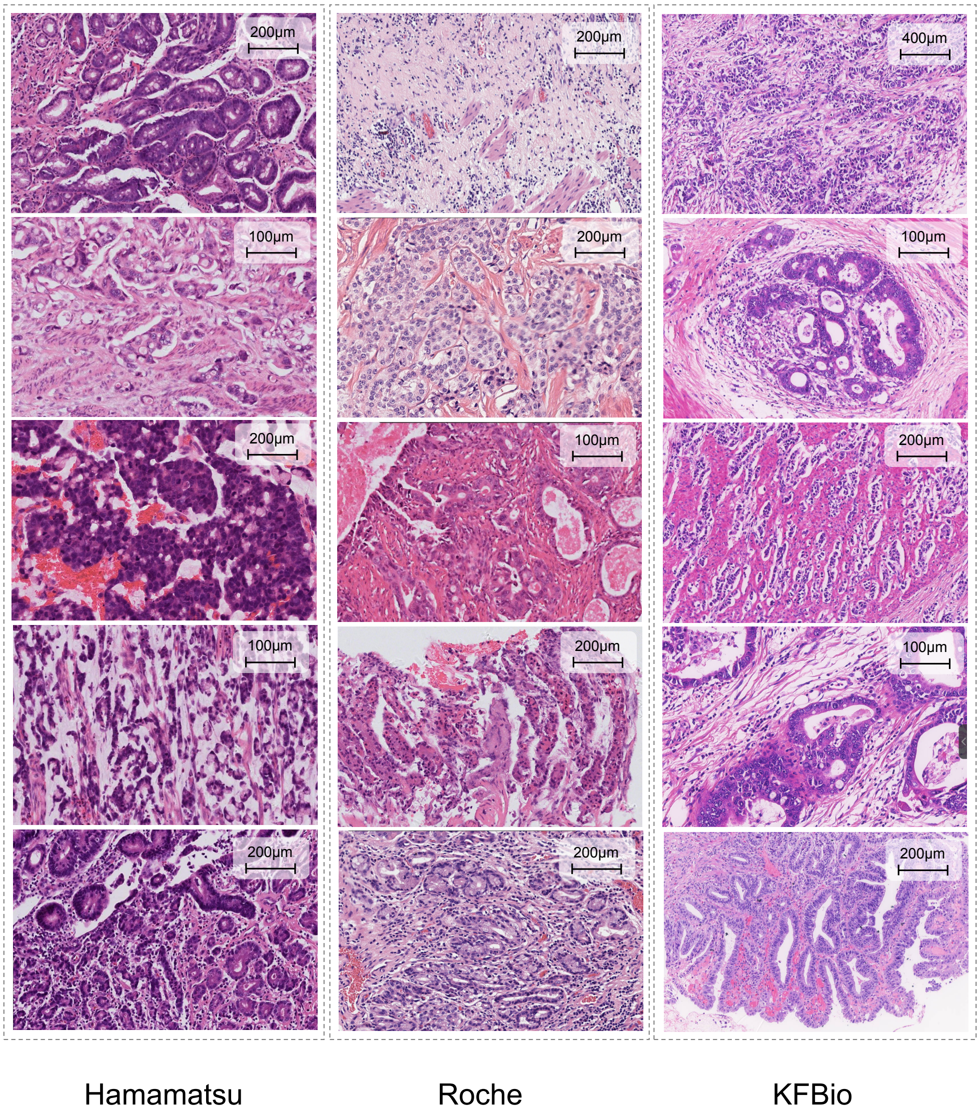

Figure 4: **Example WSIs digitalized by three different scanners from the daily gastric dataset.** All the images were captured with a 20× objective. The slides were digitalized for ten times, and we obtained similar results.

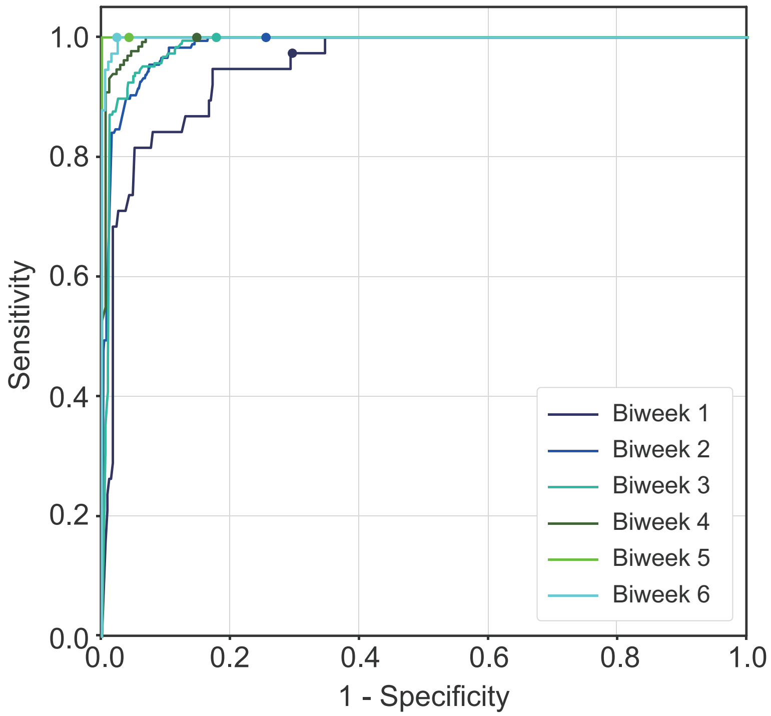

Figure 5: **Model performance on the daily gastric dataset.**

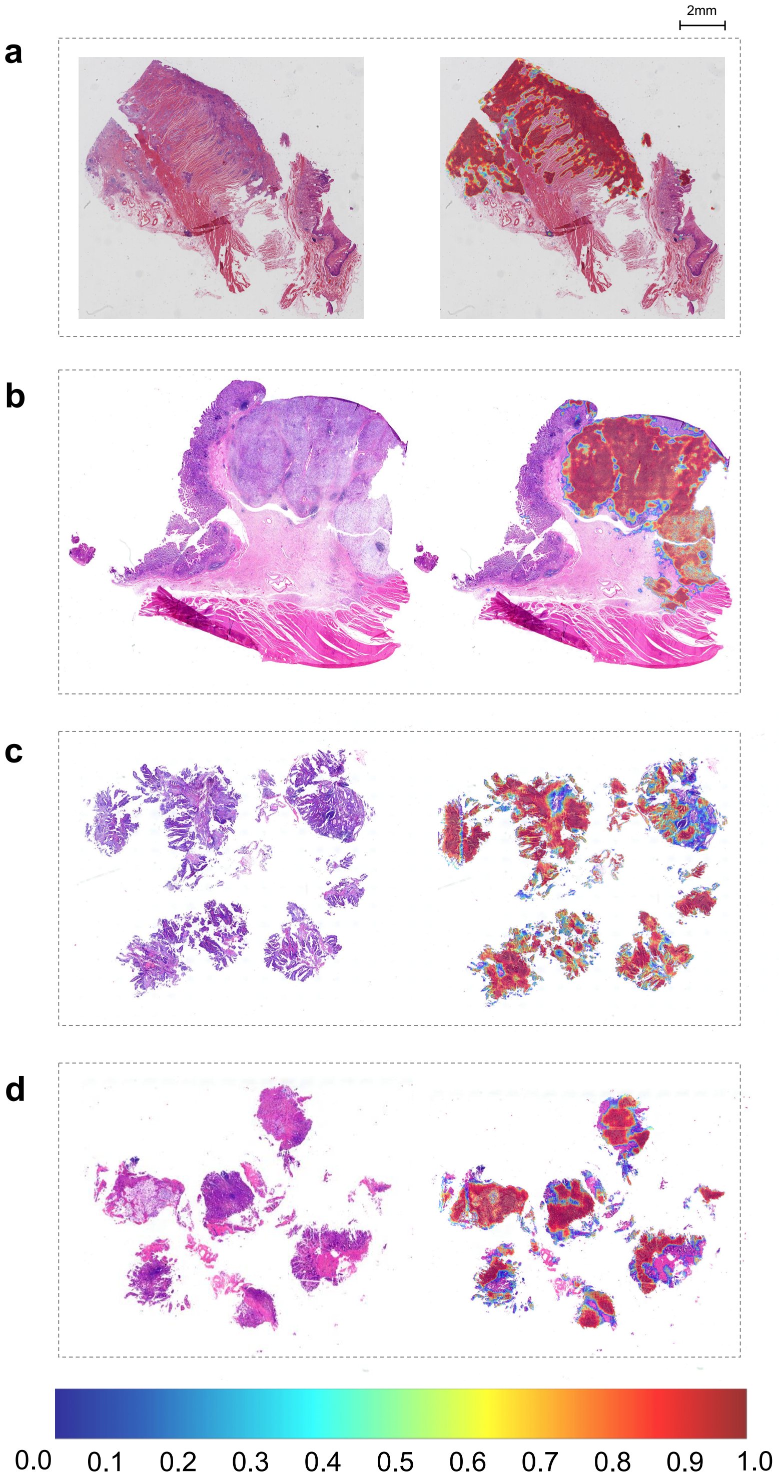

Figure 6: **Four examples of deep learning model predictions in the form of heatmaps.**

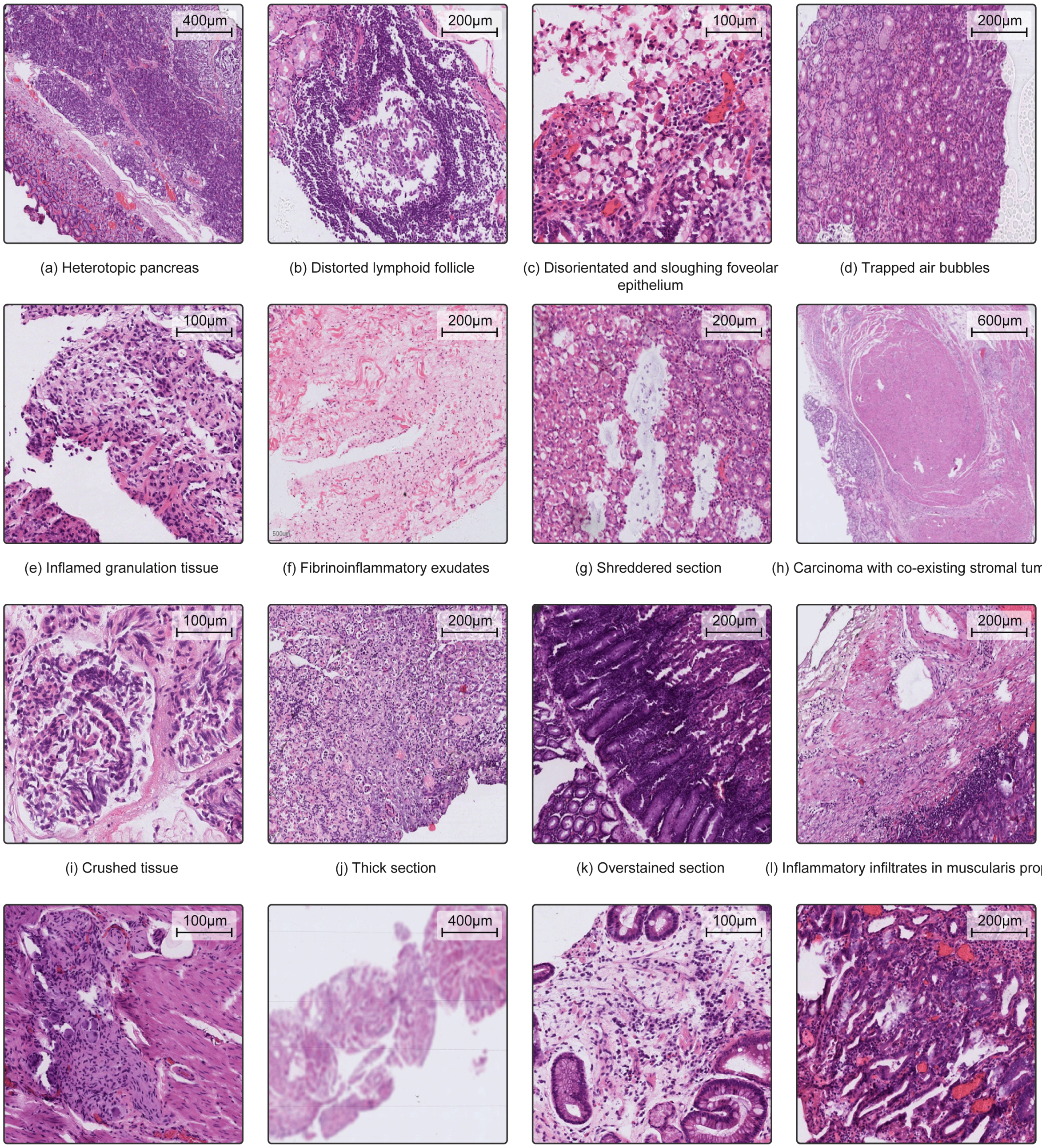

(a) Heterotopic pancreas (b) Distorted lymphoid follicle (c) Disorientated and sloughing foveolar epithelium (d) Trapped air bubbles (e) Inflamed granulation tissue (f) Fibrinoinflammatory exudates (g) Shreddered section (h) Carcinoma with co-existing stromal tumor (i) Crushed tissue (j) Thick section (k) Overstained section (l) Inflammatory infiltrates in muscularis propria (m) Hypertrophic myenteric plexus (n) Defocused section (o) Inflammatory infiltrates in edematous stroma (p) Regenerative atypia accompanied by marked inflammation

Figure 7: **More false-positive cases.** The experiment was performed five times, and we obtained the same results.

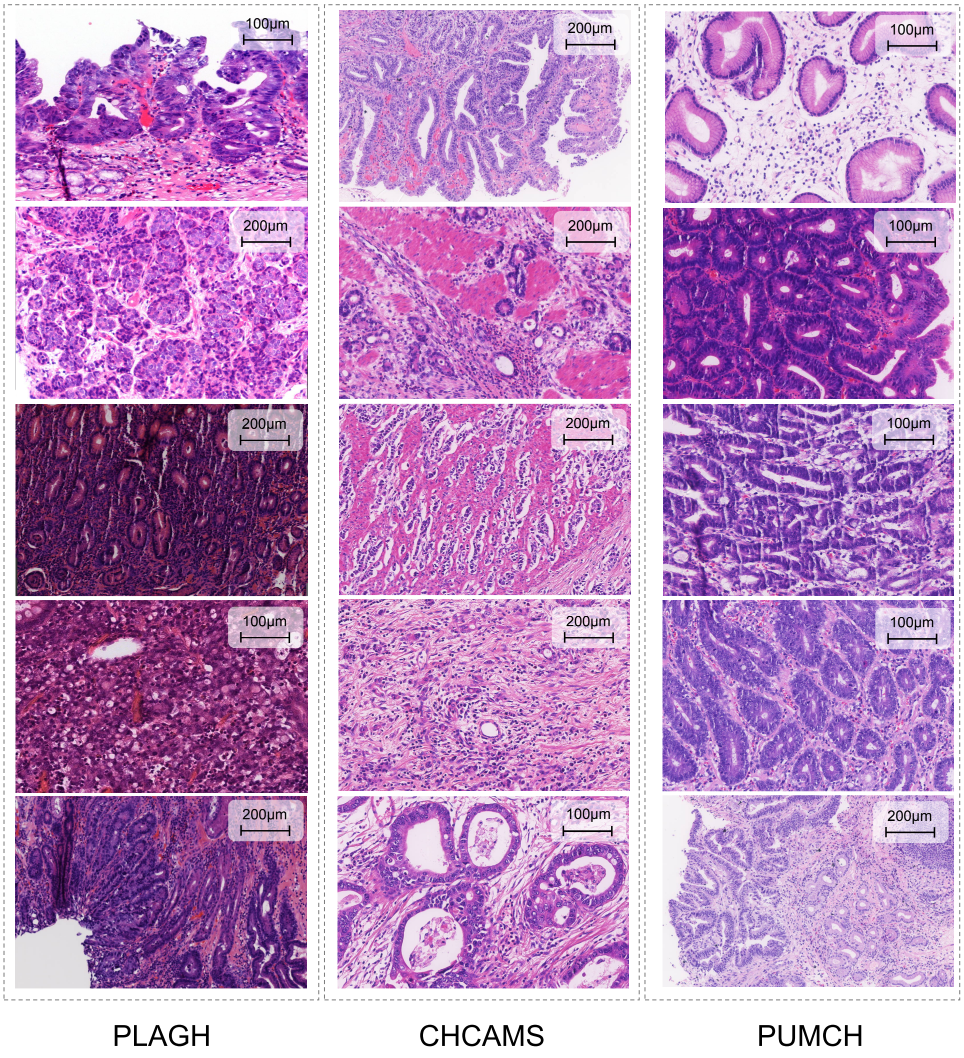

Figure 8: **Example WSIs with visual difference digitalized by KF-Pro-005 from three hospitals.** PLAGH and PUMCH used automatic H&E staining with Leica AutoStainer XL in practice, while CHCAMS adopted automatic H&E staining with Roche Ventana HE 600. Significant visual difference could be found in the images. All the images were captured with a 20× objective. The slides were prepared three times from the same blocks, and we observed similar visual appearances.

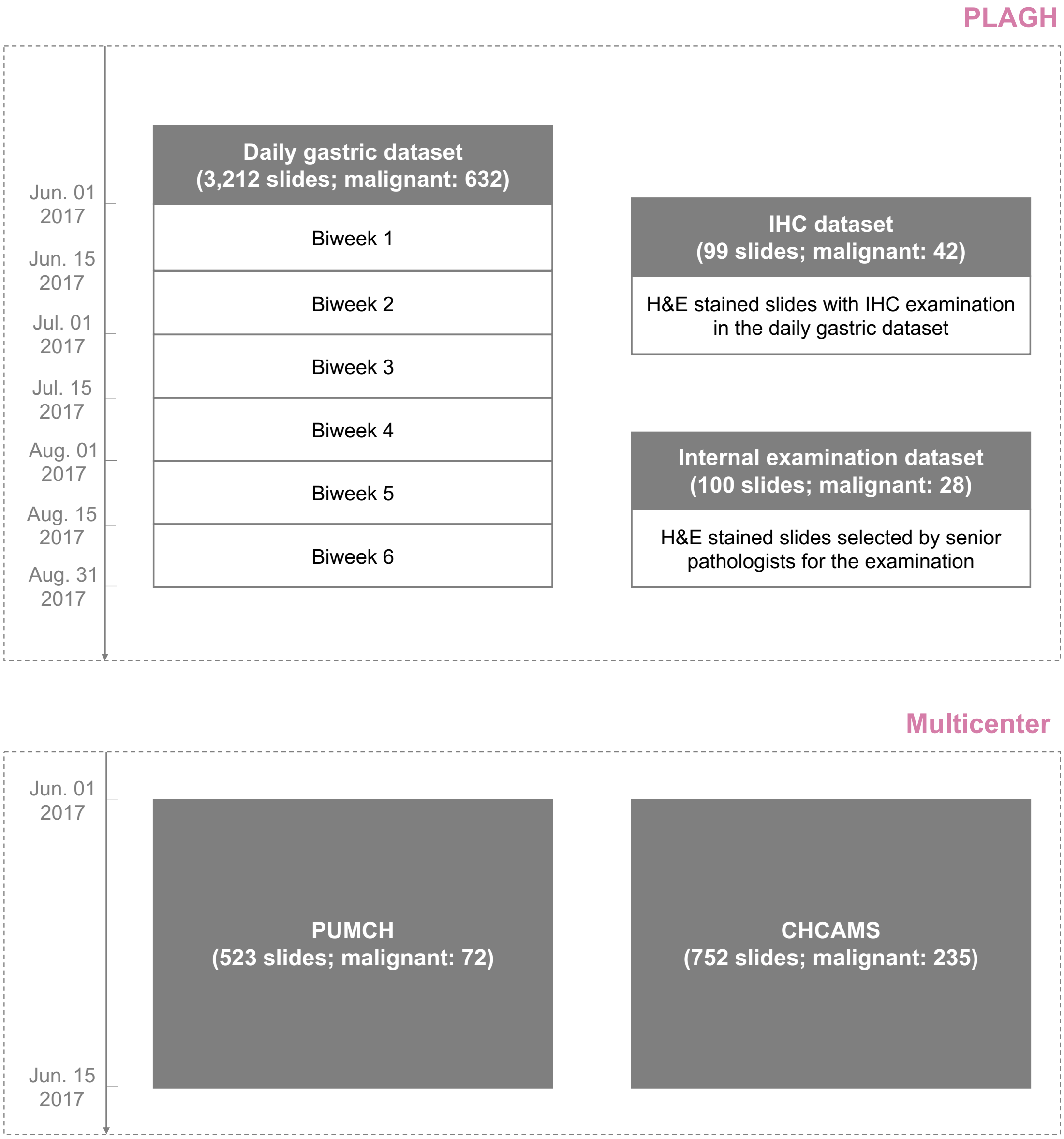

Figure 9: **Illustration of the test datasets.**

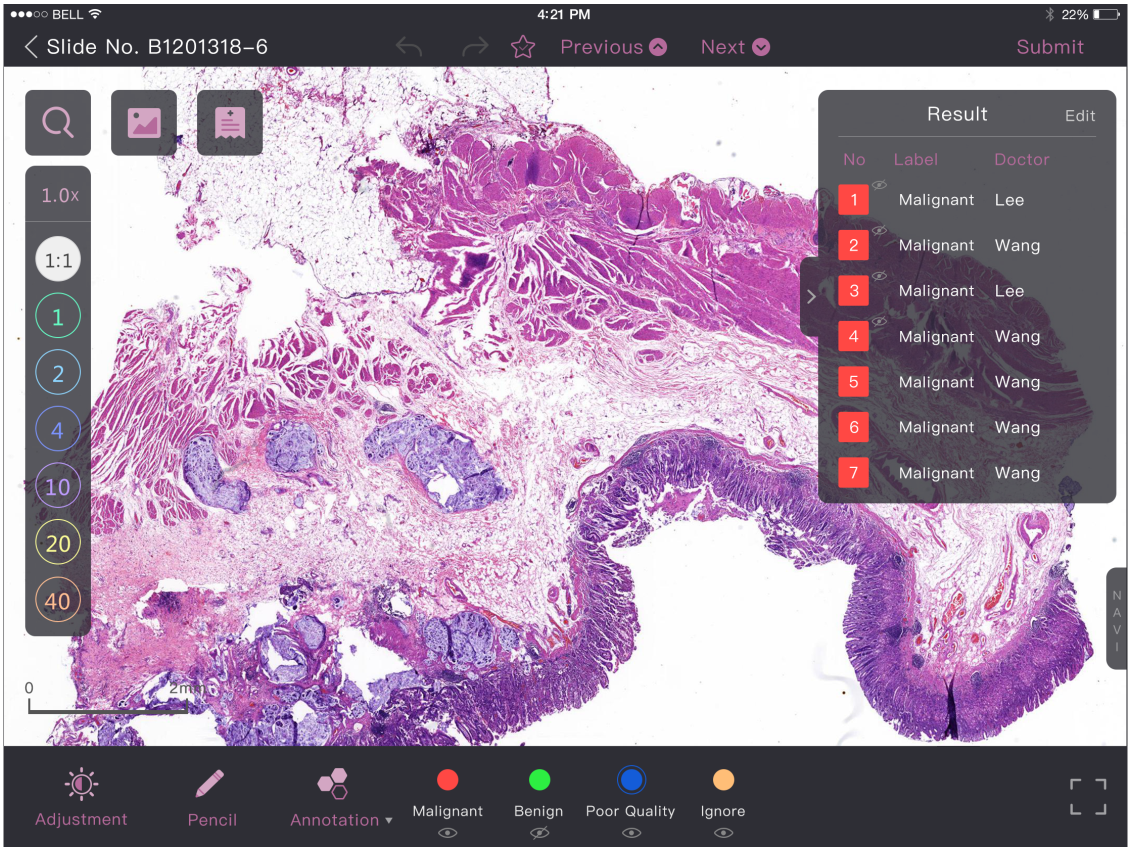

Figure 10: **iPad-based annotation system interface.**

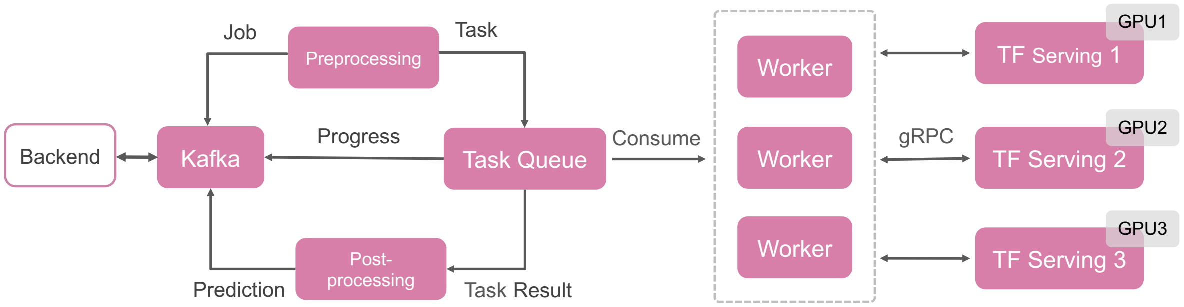

Figure 11: **AI assistance system architecture.**

GPU Number	Server Cost	Processing Capacity / 12 Hours
1	$4,000	200 Slides
2	$7,000	400 Slides
3	$11,000	600 Slides
4	$14,000	800 Slides

| Digital Scanner

Roche Ventana DP200 (6 slides): **$80,000**

Hamamatsu NanoZoomer S360 (360 slides): **$150,000**

Total Cost

Small hospital: **$84,000-$87,000**

Large hospital: **$161,000-$164,000**

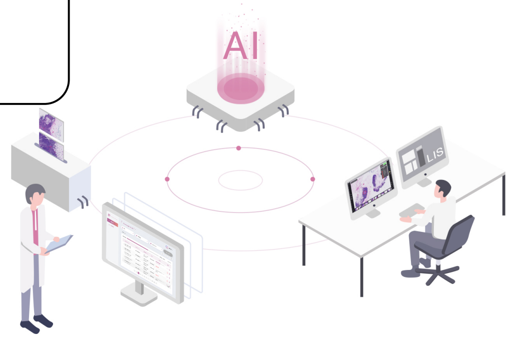

Figure 12: **A complete cost analysis of the whole assistance system.** The cost for the digital scanners were estimations of the current market price. The server hardware configuration was: [CPU] Intel Core i7, [Memory] 32GB, [Solid State Disk] 1TB, [Hard Disk Drive] 10TB, [GPU] NVIDIA Tesla P100. The cost of the diagnostic system was not included.

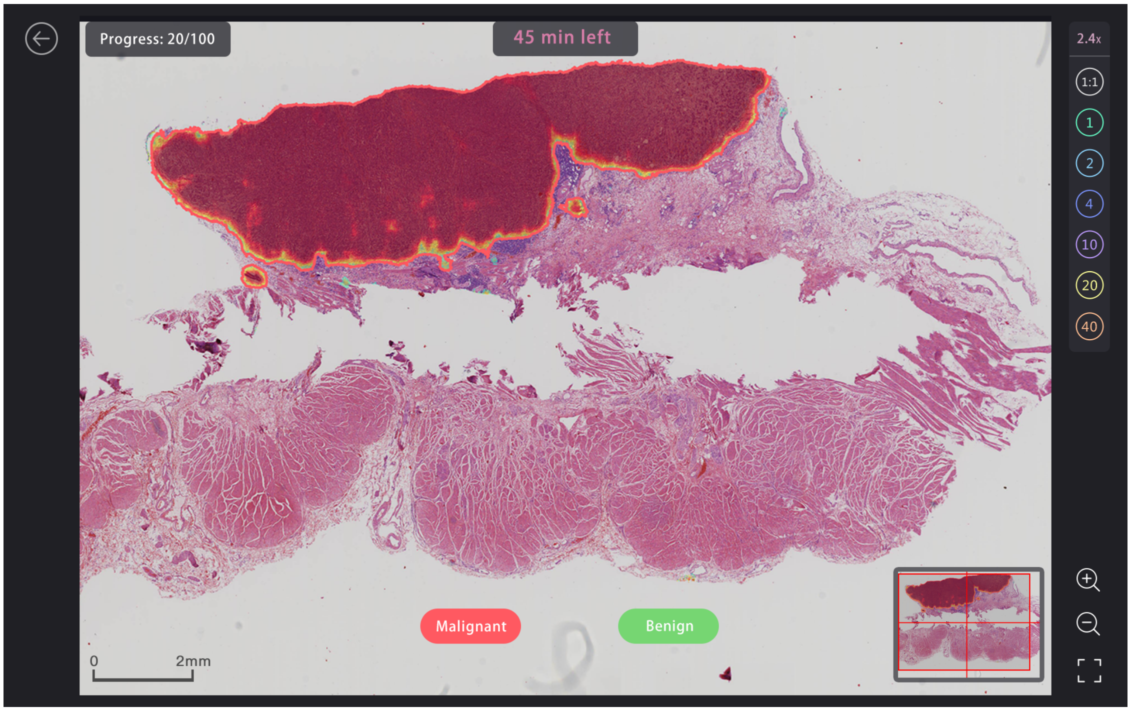

Figure 13: **The interface of the AI assistance system used in the internal examination.** The inference was performed five times, and we obtained the same result.

Table 1: **Abbreviations of tumour subtypes.**

HGIN	High grade intraepithelial neoplasia
TAC	Tubular adenocarcinoma
MucAC	Mucinous adenocarcinoma
PCC	Poorly cohesive carcinoma
MixAC	Mixed adenocarcinoma

Table 2: **Details of annotation pathologists.**

Name	Position	Education	Experience (year)	Role
Huaiyin Shi	Chief Pathologist	PhD	31	Final check
Zhigang Song	Associate Chief Pathologist	Master	16	Final check
Zhanbo Wang	Associate Chief Pathologist	Master	16	Labelling
Jing Yuan	Associate Chief Pathologist	PhD	16	Labelling
Chunkai Yu	Associate Chief Pathologist	PhD	16	Labelling
Yong Huang	Attending Pathologist	PhD	19	Labelling
Jinhong Liu	Attending Pathologist	PhD	16	Labelling
Xiaohui Ding	Attending Pathologist	Master	16	Labelling
Xin Chen	Attending Pathologist	Master	7	Labelling
Wei Jin	Attending Pathologist	Master	7	Labelling
Xiangnan Gou	Attending Pathologist	Master	7	Labelling
Liwei Shao	Attending Pathologist	Master	4	Labelling

Table 3: **Performance of different slide-level prediction approaches on the validation dataset.**

Predictor	AUC	Sensitivity	Specificity	Accuracy
Random forest	0.973	0.986	0.623	0.790
Top 10 probabilities	0.983	0.986	0.532	0.753
Top 100 probabilities	0.983	0.986	0.571	0.773
Top 200 probabilities	0.982	0.986	0.604	0.790
Top 500 probabilities	0.985	0.986	0.636	0.807
Top 1,000 probabilities	0.988	0.986	0.740	0.860
Top 2,000 probabilities	0.983	0.986	0.734	0.857
Top 5,000 probabilities	0.979	0.986	0.721	0.850
Top 10,000 probabilities	0.976	0.986	0.675	0.827

Table 4: **Performance of different classification and segmentation models for patch-level classification on the validation dataset.**

Deep learning model	AUC	Sensitivity	Specificity	Accuracy
ResNet-50	0.853	0.779	0.777	0.778
Inception v3	0.887	0.864	0.765	0.806
DenseNet	0.834	0.804	0.713	0.750
U-Net	0.779	0.950	0.369	0.550
DeepLab v2	0.884	0.918	0.710	0.775
DeepLab v3	0.945	0.944	0.780	0.833

Table 5: **Model performance on the daily gastric WSI digitalized by three scanners.**

Digital scanner	Total	Malignant	Benign	AUC	Accuracy	Sensitivity	Specificity
KF-PRO-005	403	80	323	0.995	0.950	1.0	0.938
Hamamatsu NanoZoomer S360	1832	352	1480	0.982	0.781	1.0	0.728
Ventana DP200	977	200	777	0.992	0.918	0.995	0.898

Table 6: **Pathologists' performance in the trainees' examination with one hour constraint.**

Group name	Pathologist ID	Individual performance				Group performance (average)			
		Acc.	Sen.	Spec.	Time (min)	Acc.	Sen.	Spec.	Time (min)
Microscope	6	0.820	0.929	0.778	44	0.850	0.821	0.861	53.30
	8	0.870	0.786	0.903	51				
	9	0.830	0.643	0.903	72				
	10	0.880	0.929	0.861	47				
Digital	0	0.820	0.429	0.972	48	0.845	0.696	0.903	48.25
	2	0.860	0.750	0.903	45				
	11	0.860	0.857	0.861	52				
	1	0.840	0.750	0.875	48				
AI	5	0.910	0.786	0.958	41	0.858	0.839	0.865	45.25
	4	0.850	0.786	0.875	43				
	3	0.840	0.964	0.792	47				
	7	0.830	0.821	0.833	50				

Table 7: **Pathologists' performance in the trainees' examination without time constraint.**

Group name	Pathologist ID	Individual performance				Group performance (average)			
		Acc.	Sen.	Spec.	Time (min)	Acc.	Sen.	Spec.	Time (min)
Microscope	1	0.820	0.929	0.778	60	0.810	0.911	0.771	59.25
	2	0.880	0.893	0.875	75				
	7	0.810	0.964	0.750	60				
	11	0.730	0.857	0.681	42				
Digital	8	0.820	0.821	0.819	30	0.853	0.857	0.851	40
	5	0.910	0.821	0.944	46				
	4	0.840	0.893	0.819	41				
	3	0.840	0.893	0.819	43				
AI	6	0.860	0.893	0.847	61	0.870	0.813	0.892	50.75
	9	0.840	0.679	0.903	31				
	10	0.920	0.857	0.944	53				
	0	0.860	0.821	0.875	58				

Table 8: **Distribution of benign and malignant cases and tumour subtypes in the datasets by individual patient.**

Dataset	Specimen	Benign	Malignant	Tumour subtype				
				HGIN	TAC	MucAC	PCC	MixAC
Training	Biopsy	440	102	22	59	21	0	0
	Surgical	50	908	151	579	176	6	51
Training (random forest)	Biopsy	114	32	2	21	0	2	7
	Surgical	14	52	5	42	2	1	8
Validation	Biopsy	119	60	0	27	6	13	14
	Surgical	7	86	7	51	8	12	15
Internal examination	Biopsy	68	27	3	20	0	7	0
	Surgical	4	1	0	1	0	0	0
IHC	Biopsy	30	12	0	11	0	1	0
	Surgical	1	9	0	9	0	0	0
Daily gastric (PLAGH)	Biopsy	1599	61	12	42	4	2	10
	Surgical	36	118	16	83	6	4	25
Multicentre (PUMCH)	Biopsy	330	14	5	10	0	0	0
	Surgical	3	8	0	6	0	0	2
Multicentre (CHCAMS)	Biopsy	396	59	6	51	0	3	5
	Surgical	15	71	2	49	1	3	19

Table 9: **Distribution of benign and malignant cases and tumour subtypes in the datasets by individual slide.**

Dataset	Specimen	Benign	Malignant	Tumour subtype				
				HGIN	TAC	MucAC	PCC	MixAC
Training	Biopsy	639	102	22	59	21	0	0
	Surgical	93	1,289	242	760	274	11	63
Training (random forest)	Biopsy	169	39	4	26	0	2	7
	Surgical	215	314	54	268	4	2	42
Validation	Biopsy	143	60	0	27	6	13	14
	Surgical	11	86	5	51	8	12	15
Internal examination	Biopsy	68	27	3	20	0	7	0
	Surgical	4	1	0	1	0	0	0
IHC	Biopsy	50	22	0	23	0	1	0
	Surgical	7	20	0	25	0	0	0
Daily gastric (PLAGH)	Biopsy	2,085	124	20	93	5	8	6
	Surgical	495	508	51	354	26	22	118
Multicentre (PUMCH)	Biopsy	496	30	7	24	0	0	0
	Surgical	27	42	0	38	0	0	4
Multicentre (CHCAMS)	Biopsy	685	81	20	68	0	5	8
	Surgical	67	154	6	105	3	6	43

Table 10: **The detailed training configurations for different deep learning models.**

Deep learning model	Training iteration	Batch size	Learning rate	Decay step (by 0.5)
ResNet-50	145,000	10×4	2×10^{-3}	20,000
Inception v3	51,000	32×4	1×10^{-3}	10,000
DenseNet	165,000	20×4	2×10^{-3}	20,000
U-Net	75,000	20×4	2×10^{-3}	20,000
DeepLab v2	189,000	10×4	2×10^{-3}	20,000
DeepLab v3	95,000	32×4	1×10^{-3}	20,000

Table 11: **Features used for the random forest model.** The eccentricity, extend, major axis length, and solidity are defined as ellipse with the same second moment, ratio of the region area over the bounding box, length of the major axis of the ellipse with the same normalized second central moment, and ratio of the region area over the surrounding convex, respectively.

Number	Feature definition
1-5	Ratios of cancer to tissue (thresholds: 0.5, 0.6, 0.7, 0.8, 0.9)
6-10	Ratios of probability sum of cancer to tissue (thresholds: 0.5, 0.6, 0.7, 0.8, 0.9)
11-14	Largest area, eccentricity, extend, and bounding box area
15	Major axis length
16-17	Maximum/minimum probability in the region
18	Largest mean probability in the region
19	Aspect ratio of the bounding box
20	Solidity
21-24	Second largest area, eccentricity, extend, and bounding box area
25	Minor axis length
26-27	Second maximum/minimum probability in the region
28	Second largest mean probability in the region
29	Aspect ratio of the bounding box (second largest)
30	Second largest solidity
