# Supplementary material for: Clinically applicable histopathological diagnosis system for gastric cancer detection using deep learning
Source: Nat Commun. 2020 Aug 27;11:4294. doi: 10.1038/s41467-020-18147-8 (PMC7453200; doi:10.1038/s41467-020-18147-8)
Supplement: Supplementary file 3 — Reporting Summary [file 41467_2020_18147_MOESM3_ESM.pdf]

nature research

Corresponding author(s): Shuhao Wang and Huaiyin Shi

Last updated by author(s): Jul 29, 2020

Reporting Summary

Nature Research wishes to improve the reproducibility of the work that we publish. This form provides structure for consistency and transparency in reporting. For further information on Nature Research policies, see Authors & Referees and the Editorial Policy Checklist .

Statistics

For all statistical analyses, confirm that the following items are present in the figure legend, table legend, main text, or Methods section.

n/a	Confirmed	
☐	☒	The exact sample size (*n*) for each experimental group/condition, given as a discrete number and unit of measurement
☐	☒	A statement on whether measurements were taken from distinct samples or whether the same sample was measured repeatedly
☒	☐	The statistical test(s) used AND whether they are one- or two-sided<br>*Only common tests should be described solely by name; describe more complex techniques in the Methods section.*
☒	☐	A description of all covariates tested
☒	☐	A description of any assumptions or corrections, such as tests of normality and adjustment for multiple comparisons
☐	☒	A full description of the statistical parameters including central tendency (e.g. means) or other basic estimates (e.g. regression coefficient) AND variation (e.g. standard deviation) or associated estimates of uncertainty (e.g. confidence intervals)
☒	☐	For null hypothesis testing, the test statistic (e.g. *F*, *t*, *r*) with confidence intervals, effect sizes, degrees of freedom and *P* value noted<br>*Give P values as exact values whenever suitable.*
☒	☐	For Bayesian analysis, information on the choice of priors and Markov chain Monte Carlo settings
☒	☐	For hierarchical and complex designs, identification of the appropriate level for tests and full reporting of outcomes
☒	☐	Estimates of effect sizes (e.g. Cohen's *d*, Pearson's *r*), indicating how they were calculated

Our web collection on statistics for biologists contains articles on many of the points above.

Software and code

Policy information about availability of computer code

Data collection	Glass slides were digitized with KFBio KF-Pro-005, Hamamatsu NanoZoomer S360, and Roche Ventana DP200 at 40x magnification.
Data analysis	The code was written in Python 2.7. All the plots were made using the matplotlib package in Python. We used ThoSlide 2.1.0, a proprietary library, to access the whole slide images, and TensorFlow 1.10.0 to train deep learning models. We built our deep learning model based on DeepLab v3 with the ResNet-50 architecture as its backbone. TensorFlow Serving 1.3.0 was used to serve the model as a service at inference time. The scheduler was realized using Celery 4.3.0. We used Kafka 2.1.0 as the message queue.<br>The training code base for the deep learning framework is available at: https://github.com/ThoroughImages/NetFrame. This framework is general and can be applied to other organs. The core components of the inference system are available at: https://github.com/ThoroughImages/PathologyGo.

For manuscripts utilizing custom algorithms or software that are central to the research but not yet described in published literature, software must be made available to editors/reviewers. We strongly encourage code deposition in a community repository (e.g. GitHub). See the Nature Research guidelines for submitting code & software for further information.

Data

Policy information about availability of data

All manuscripts must include a data availability statement. This statement should provide the following information, where applicable:

- Accession codes, unique identifiers, or web links for publicly available datasets
- A list of figures that have associated raw data
- A description of any restrictions on data availability

The data that support the findings of this study are available on request from the corresponding authors (H.S. and S.W.). The data are not publicly available due to hospital regulations.

Field-specific reporting

Please select the one below that is the best fit for your research. If you are not sure, read the appropriate sections before making your selection.

- [x] Life sciences
- [ ] Behavioural & social sciences
- [ ] Ecological, evolutionary & environmental sciences

For a reference copy of the document with all sections, see nature.com/documents/nr-reporting-summary-flat.pdf

Life sciences study design

All studies must disclose on these points even when the disclosure is negative.

Sample size	The sample size was determined by the number of slides we could digitize in the three hospitals. The daily gastric dataset contains 1,814 cases (3,212 WSIs) from PLAGH. The multicentre dataset contains 355 cases (595 WSIs) from PUMCH and 541 cases (987 WSIs) from CHCAMS. In summary, the two datasets comprise 4,794 WSIs. The datasets used in this research covers various subtypes of gastric cancer, sufficient for the model training and testing.
Data exclusions	No exclusion
Replication	We replicated the high performance of our model on the multicentre dataset, which contains WSI data from two different hospitals, prepared with different stainer models and digitized with different scanner models. All the results were the same during the replication.
Randomization	Datasets were randomly assigned to different sets (training, validation, and test).
Blinding	Since our experiments were based on WSIs with no patient information, blinding was not necessary.

Reporting for specific materials, systems and methods

We require information from authors about some types of materials, experimental systems and methods used in many studies. Here, indicate whether each material, system or method listed is relevant to your study. If you are not sure if a list item applies to your research, read the appropriate section before selecting a response.

Materials & experimental systems

n/a	Involved in the study	
☒	☐	Antibodies
☒	☐	Eukaryotic cell lines
☒	☐	Palaeontology
☒	☐	Animals and other organisms
☒	☐	Human research participants
☒	☐	Clinical data

Methods

n/a	Involved in the study	
☒	☐	ChIP-seq
☒	☐	Flow cytometry
☒	☐	MRI-based neuroimaging
